# Exploring dialogue in virtual simulation in nursing education – An observational study^☆^

**DOI:** 10.1016/j.pecinn.2024.100294

**Published:** 2024-05-24

**Authors:** Maarten van der Vloed, Hilde Eide, Lise Gladhus, Kirsten Røland Byermoen, Hugrun Ösp Egilsdottir, Lena Günterberg Heyn

**Affiliations:** a Center for Health and Technology, University of South-Eastern Norway, Campus Drammen, Grønland 58, Drammen 3045, Norway; b Ede Christian University of applied sciences, Oude Kerkweg JS Ede, Netherlands

## Abstract

**Objectives:**

Simulation is an important learning activity in nursing education. There is little knowledge about dialogue and communication between students and facilitators in a virtual simulation setting. The current study, conducted in Norway, explores the dialogic teaching approaches applied by facilitators in a virtual classroom and adapt an analytic tool from a physical classroom in lower education to a virtual classroom in higher education.

**Methods:**

Sixteen virtual simulation sessions of groups with nursing students were video-taped. The videos were coded with a coding scheme developed for physical classrooms and adapted to the virtual setting. The dialogic approaches from the facilitator were analysed using descriptive analysis.

**Results:**

The most frequently used approaches from the facilitator were categorised as asking (“Big questions”) and listening (“Wait time after a question”). The most frequent pattern seen in the use of dialogic approaches fall under the category listening.

**Conclusions:**

The coding scheme is suitable to analyse facilitators' dialogic approaches in a virtual setting in nursing education. Further research should examine how the facilitator can strategically deploy dialogic approaches in other types of simulations with students.

**Innovation:**

The coding scheme was developed from lower to higher education, and from a physical to a virtual setting.

## Highlights

• We adapted an analytic tool to study facilitation in a virtual simulation setting.
• Facilitators in virtual simulation use approaches for dialogic teaching.
• Facilitators use the approaches “ask” and “wait” more than the approach “listen”.

## 1 Introduction

Introducing technology into higher education creates new opportunities for teaching and learning. In nursing education, low- and high-fidelity simulation are well-established learning activities [1]. More recently, simulation training has also become more technological with software offering virtual learning experiences. Implementing new technology, such as virtual simulation, opens up opportunities to explore communication and dialogue between faculty members and students [[2], [3], [4]].

In nursing education, a facilitator is present in the simulation sessions to guide the students towards their learning goals. The possible role of the facilitator is described in a recent review as an important aspect of the learning [5]. This review included studies from 15 different countries, where the simulation approaches were similar internationally. The facilitator can help students thrive in the simulation, strive to learn more, improve their thinking process, learn to listen to fellow students' opinions, and have a closer connection with their facilitators [6]. The digital transformation, challenges faculty members to reflect on how to structure learning activities that are beneficial for students' learning [7]. This shifts the facilitator's perspective from deciding what students do, to engage in dialogue with students about how they best learn and what they need to build an effective learning environment. Dialogue and feedback are the most important strategies for guiding students in a learning process and helping to promote their development and competence [8,9]. The facilitator supports students in acquiring knowledge and developing skills and stimulates to reach an even deeper level of reflection by asking questions [10] and, most important, waiting to hear their answers, which means leaving room for silence [11].

Communication and dialogue in groups has been the subject of educational research for many decades with a specific focus on the concept of dialogic teaching [[12], [13], [14]]. In dialogic teaching, discussion is used to create interest, encourage thinking, promote understanding, share ideas, and develop and assess arguments, empowering students to engage in learning [12]. When a facilitator is supportive and stimulates students to be collaborative, this creates social and emotional engangement. Dialogic teaching encourages students to voice their ideas and thoughts and enables facilitators to diagnose students' needs, craft learning assignments, increase understanding, assess progress, and guide students through the challenges they face. Dialogue positively affects learning by triggering processes that contribute to the development of knowledge, and studies show that dialogic teaching result in better learning than more traditional teacher-led teaching [15,16]. Dialogue creates free access or flow of meaning between people communicating with each other, and students who are stimulated to talk more, will also learn more [17,18].

By entering into dialogue with students, facilitators can change the dynamics of the learning situation [15]. Bringing dialogic teaching into a simulation setting, implies the facilitator invites students to talk rather than providing as much information as possible [19,20]. This dialogue creates an environment in which facilitators and students can disagree and question and challenge each other and the facilitator can also challenge students to dialogue with each other. Hence, the focus shifts from the facilitator to the whole group, thus transforming the learning environment into a place where learning is celebrated [19].

Virtual simulations are different than in-person simulations, and there is a lack of knowledge about how facilitation needs to adjust to the digital format to ensure learning. During virtual simulation, students are not physically present in a room where the facilitator can observe them talking to each other and class management works differently than in an in-person simulation. The details about how this affects the facilitators' approaches to stimulate reflection and learning is not known.

### 1.1 Aim of the study

The aim of this study was twofold: 1) to adapt an analytic tool used in a traditional classroom to a digital learning environment in higher education, and 2) to explore what dialogic teaching approaches a facilitator applies to stimulate dialogue with or among nursing students in a virtual simulation in a digital learning environment.

## 2 Method

### 2.1 Research design

This is an observational study, conducted in Norway, of video-recorded virtual simulations that were mandatory for bachelor nursing students in their first year.

### 2.2 Sample and procedure

This research was conducted at a large Norwegian university during the COVID-19 lockdown, and the data was collected during a 10-week period between April and June 2020. The first-year nursing students participating in the study had not had any clinical rotation in their first year of education. The distribution of gender and age among the students is not known as no demographic data were collected.

The students participated in a digital clinical course due to the pandemic restrictions as described by Egilsdottir and colleagues [21]. One of the learning activities in the digital clinical course was virtual simulation sessions conducted in a commercial video conference system (CVCS). A total of 43 students were divided into groups of seven to nine students, and the same groups simulated together four times during the digital clinical course. Altogether, 33 students were present in all meetings. Twelve recorded virtual simulation sessions were available for analysis and included in this study. Two faculty members were present in the simulation sessions: one main facilitator responsible for interacting with the students and handling the software and a co-facilitator who helped stimulate students' reflections when needed. The main facilitator was the same person in all the virtual simulation sessions. Both facilitators worked at the university and had no prior experience with dialogic teaching or conducting virtual simulation in a CVCS.

### 2.3 The virtual simulation setting

A virtual patient was presented to the students, and the main facilitator interacted with the students to solve the case. A simulation software, Body Interact™ (www.bodyinteract.com), was used to access virtual patients. Body Interact™ has a variety of virtual patient cases and a physiological algorithm that creates dynamic clinical situations in which the virtual patient responds to the user's actions and interventions or lack of thereof. The students were challenged to map the virtual patient's clinical situation, collect subjective (communication) and objective (physical assessment) data needed to formulate appropriate nursing diagnoses. The virtual patient cases represented common clinical problems related to different organ systems (see Table 1).

**Table 1:** An overview of the virtual patient cases used in the group simulations.

	Respiratory System	Circulation system	Neurological system	Abdominal system
ABCED assessment	**A & B** Airway and breathing	**C** Circulation	**D** Disability	**E** Exposure
Clinical problems in the different virtual patient cases	Ineffective breathing due to COPD exacerbation	Impaired circulation due to heart failure	Ineffective cerebral tissue perfusion due to stroke	Impaired effective elimination due to urinary retention

As the students were also learning to conduct the ABCDE (airway, breathing, circulation, disability and exposure) assessments the chosen virtual patient case was related to clinical problems representing each area of assessment (Table 1) The students could also interact with the virtual patient through predetermined dialogues in the software, i.e. asking questions to the patient about the medical condition, about medications, risk factors and physical activity, who would give a response. Since the virtual simulation was conducted on Zoom, the main facilitator shared her screen and was responsible for executing the actions in the virtual patient case suggested by the students. When the virtual simulation started, the main facilitator presented the virtual patient case and the learning outcomes. Thereafter she interacted with the students, challenging them as the case proceeded to verbally formulate their reasons for choosing different actions. This means that the focus of the virtual patient case was not just ‘playing the game’, but also to justify one's actions by articulating verbally own professional knowledge. The students' actions included asking appropriate questions, choosing which vital signs to assess, conducting a physical assessment, or performing nursing interventions. At the end of the virtual simulation, the main facilitator showed the Body Interact feedback summary presenting the results of the students' diagnoses and treatments. The feedback summary displayed the performed physical examinations, the diagnostic activity and treatment during the simulation, and provided the students with a global score. These were then reflected on in the group. Finally, the faculty evaluated the group process in the virtual simulation.

### 2.4 **Coding scheme –** Dialogic approaches coding scheme (DACS)

A modified version of the teaching approaches for dialogue in traditional classroom was used. Content of this website is available under the Creative Commons Attribution-Share Alike or Attribution-Non-Commercial licence, which means that anyone is free to share and to adapt material from this open source [22]. The name of this scheme, Features of Effective Dialogue, was changed in this study to Dialogic Approaches Coding Scheme (DACS). The coding scheme allows for systematic observation of specific approaches to dialogic teaching used by the facilitator in a student group. There are 15 teaching approaches in dialogic teaching, for conducting dialogue in a group of students, see Table 2.

**Table 2:** Teaching / dialogic approaches.

Number	Dialogic approaches	Description
1	Eavesdropping on group dialogue	Facilitators look for evidence of learning by listening, either to transfer ideas from one group to another or to feed the ongoing conversation/dialogue within the entire group at a later time. Facilitators use the time to listen to students and not get involved in the conversation. They will return to what they have heard later. Facilitators can determine in advance the order in which feedback is given within groups to encourage a rich discussion within the entire group. Sometimes they get involved in the dialogue to encourage a more effective group conversation. In this case, they give direction to students' conversation.
2	Questions linked to resources or tasks	The facilitator uses resources to help introduce an issue through a specific question. Resources can be powerful aids if they are chosen to set up and complement both challenging questioning and learning through responses to the challenges. Think of a book/article/website. Or the facilitator can respond to a source that a student contributes by rating it and discussing it with the group. The facilitator can also actively ask students to contribute a source.
3	Wait time after a facilitator question	Students are given time to reflect independently on a question and to think and formulate their ideas and construct a response before being asked to answer. The facilitator may make this explicit by saying that they can think for a while. This requires at least three seconds.
4	Big questions	These are significant questions that cannot be answered immediately. By its nature, a big question draws answers from many students and encourages them to come up with a list of smaller questions they need to answer before an answer to the big question can be formulated. Sometimes the smaller questions are provided by the facilitator – questions that call for other questions and the will to research. The facilitator is not really looking for the right answer. Closed questions that only require yes or no do not belong to in this category.
5	Acknowledge when students demonstrate effective dialogue	Facilitators explicitly comment on the features of effective dialogue where they occur. The facilitator appreciates what the student does to sustain the conversation and makes that explicit.
6	Pausing to scan or survey	Facilitators stand back to take stock of the learning across the class. This enables them to assess quickly what the students can do, can partially do or cannot do, to hear the language students are using as they work with others and to adjust their teaching in response. They summarize where they think students stand. You hear/see that the facilitator adjusts their plan.
7	Wait time after a student's response	Students are given time to reflect on a peer's response to a question. This enables them to check whether they understand it and to formulate a further response that builds on what has been said. This requires at least three seconds.
8	Using incorrect or partially correct answers to prompt responses	Facilitators model not being sure about what the right answer is: Facilitators are seen to take risks and be vulnerable, or they help students unpack thinking leading up to partially correct response and get others to challenge or support each step. Facilitators make their thought process explicit and mirror it back to the student.
9	Rich questions	These are open-ended, higher-order questions which require learners to either link or apply ideas, give reasons, summarize, or evaluate. The facilitator summarizes and then asks what this means or what the students see in concrete terms. Sometimes they encourage students to ask themselves further questions to qualify what the question is asking them to explain. Such questions generally require extended answers. Closed questions which only can be answered with yes or no are not included in this section.
10	Modelling prompts and body language to encourage continuation	Facilitators use body language or oral prompts to encourage students to develop their answers, for example ‘Go on…’ or nodding when the student stalls. Also, the facilitator can ask another student to elaborate on the answer of a fellow student. By making these prompts explicit, the intention is that students will then adopt similar strategies in their group dialogue.
11	No-hands-up questioning	Facilitators select the student who will respond to a question. By watching students' body language, it is often possible to identify those who have ideas to contribute. The facilitator explicitly says that they can see that the student is busy formulating an answer and ask the student if that observation is correct.
12	Peer discussion	Facilitators prompt dialogue, often via a question, to enable peer interaction to support learning. The opportunity to discuss ideas in pairs or small groups (which may be with peers who share the same first language) helps students articulate and check ideas before they reveal their group's answer to the whole class. Answers are formed more easily through group discussion. The facilitator moves towards the class in order to create dialogue.
13	Modelling interest and enthusiasm	Facilitators model respect for others' points of view by reflecting on them and exploring them or they model a positive response to sincere ‘off the wall’ comments or show excitement about a good response. The facilitator is explicitly non-verbal or verbal in their enthusiasm, for example saying, “great that you say this” or “impressive that you come to this conclusion”.
14	Varying length of wait time	The length of wait time is adjusted according to the importance of the question and how challenging it is, for example from a few seconds for thought to longer pauses of a few minutes for reflection or discussion. This will take at least three seconds.
15	Negotiating whether answers are right or incorrect and why	Facilitators invite a vote on a reasoned response, crystallize the views of two camps to help focus further discussion, or constructively challenge points raised by providing an alternative argument or perspective. Facilitators will say for example, “Can you be more precise in your answer, or is this your final answer?”

### 2.5 Data analyses

The 12 videos lasted between 47 and 72 min. All videos recorded the virtual simulation sessions from the beginning to the end of the sessions. The researchers watched all the videos multiple times, both individually and together. The first step in the analysis process was to watch the videos together (MV, LGH, LG, HE) without coding or using the measurement instrument. The second step was for each researcher to watch the same video individually and code the dialogue techniques used by the facilitator using the coding scheme (DACS). The project group, which included experts in communication skills training and research and nursing educators, coded in consensus first until agreement was satisfactory. Two members (MV and LG) coded the data and validated with the whole group when needed.

An iterative analytical process provided new insights for interpreting how the main facilitator interacted with the students and was integrated into the way the coding was conducted. We observed that the facilitator used different approaches than those captured by the existing codes to obtain dialogue. There was a consensus that the facilitator did two additional things. First, it was observed that the code “Questions linked to resources or tasks” did not cover everything that happened in the virtual context. We decided to add a sub-code for when the facilitator used the virtual simulation program as a source for a question. Second, the facilitator exhibited an additional behaviour in the dialogue, which we called “class management”. We added the sub-code 2.4 (questions in relation to the Body Interact™ program) and code 16 (class management), with the sub-codes 1 to 4 (1: Translates to English or Norwegian, 2: Corrects students, 3: General information, and 4: Teacher gives info/answer/lecture).

After the additional codes were added to capture the difference between traditional classroom and virtual classroom, the videos were coded from the beginning until the end using Excel. A part of the coded fragments was provided with a memo to make it possible to find the trade-offs in coding at a later stage. To support transparency and credibility, a log was kept in which the considerations and choices made were noted during the study. The results were presented to the main facilitator for validation.

We performed descriptive analysis of the approaches used. To illustrate specifically what the facilitator did, we identified the three main dialogic teaching skills initiated by the facilitator during the virtual simulation sessions: *asking, listening,* and *waiting* (Table 3). The approaches used in dialogic teaching seem to fall into these three main skills. This categorization provided in-depth description of the results.

**Table 3:** Sixteen approaches in dialogic teaching categorised by three main dialogical skills.

To ask	To listen	To wait
Questions linked to resources or tasks	Eavesdropping on group dialogue	Wait time after a facilitator question
Big questions	Acknowledge when students demonstrate dialogue	Pausing to scan or survey
Using incorrect or partially correct answers to prompt responses	Modelling prompts and body language to encourage continuation	Wait time after a student's response
Negotiating whether answers are right or incorrect and why	Modelling interest and enthusiasm	Varying length of wait time after a student asked a question
No-hands-up questioning	Class management	
Peer discussion		
Rich questions		

We were interested to see if there were patterns in the approaches used by the facilitator. Hence, we identified which approaches were used in proximity to each other.

### 2.6 Research ethics

The Dean of Faculty of Health and Social Sciences at the University of South-Eastern Norway campus Drammen gave permission for the study. The Norwegian Centre for Research Data (NSD) approved the study (Ref. nr. 674,624). The study can be classified as an educational exploration: no patients were involved, and therefore no ethics committee approval was required. The facilitator orally informed department administrators before the students' clinical practice. The students were informed of the study; they provided written consent and were free to withdraw at any time. Participation had no effect on the student's clinical course. Each simulation session started with the facilitator reminding the students about the recording. As this was conducted in a CVCS, every participant had to manually consent to videorecording to stay in the session. Consent was thus given several times, both by students and facilitators. The use of camera was optional, and the students had the option to comment in the chat rather than talk on camera if they preferred. The video files and the consent the students gave were stored securely, and the data were deidentified. One of the researchers was the facilitator in the virtual clinical course but was not involved in the analysis of the video files. The researcher participated in dialogues regarding the verification of the results of the analysis.

## 3 Results

Table 4 shows the approaches that facilitators used by category in all 12 virtual simulations. All 16 approaches were used in the 12 virtual simulation sessions. The category “Modelling prompts and body language to encourage continuation” (approach *listen*) was used in all the sessions and was used more often than the others (Table 5). The most frequently used categories under the approaches *ask* and *wait* were “big questions” and “wait time after facilitator question”, respectively.

**Table 4:** Summary of used approaches per group.

Code		Group 1 4 simulations	Group 2 4 simulations	Group 3 2 simulations	Group 4 2 simulations	Total 12 simulations
To ask						
4	Big questions	199	263	133	88	683
2	Questions linked to resources or tasks	146	163	121	84	514
11	No-hands-up questioning	65	97	49	37	248
9	Rich questions	35	52	22	19	128
15	Negotiating whether answers are right or incorrect and why	41	15	5	5	66
8	Using incorrect or partially correct answers to prompt responses	13	21	16	9	59
12	Peer discussion	1	0	0	0	1

To listen						
10	Modelling prompts and body language to encourage continuation	346	408	205	227	1186
13	Modelling interest and enthusiasm	97	183	114	57	451
1	Eavesdropping on group dialogue	1	6	3	10	20
5	Acknowledge when students demonstrate effective dialogue	1	5	1	2	9
16	Classroom management	310	319	181	109	917

To wait						
3	Wait time after a facilitator question	69	84	37	49	239
14	Varying length of wait time	26	34	21	17	100
6	Pausing to scan or survey	17	8	7	14	46
7	Wait time after a student's response	2	1	0	5	8

**Table 5:** Illustration of approaches with quotation.

Code nr.	Approach	Quotes / observed behaviour (video nr.)
10	Modelling prompts and body language to encourage continuation	The facilitator nods yes and says: “Yeah……. that's right.” (1–1) The facilitator nods yes and says: “What did you say?” (2–2)
4	Big questions	Are you thinking of the normal temperature? (3–1) How can this be bad for a patient with heart problems? (2–2)
2	Questions linked to resources or tasks	Did something happen here with the vital signs? (3–2) Now we take it with us. And then I'm very curious about what you see in this picture right here. (4–2)
13	Modelling interest and enthusiasm	The facilitator smiles and says: “Very good, good question!” (1–3) Yes, but then we're on to what *she* just talked about, very well! (2–3)
11	No-hands-up questioning	«And then I immediately wonder, because we are starting the program, what do you think about that BMI there?» The facilitator waits and looks at the screen. (4–4) «If we elaborate a little on what you say there then. Do you remember which communication tool we can use if we were to call the doctor in relation to this situation? Let's say that Paul is in a nursing home.» (3–1)
16	Class management	“I'm going to put in the chat here now that link to the online form after the simulation, then stop the recording… and stop sharing the screen, then I'll look straight ahead and straight at you.” (1–1) “You [name], do you think that everything that is written here is ok for you? “(2–4)
9	Rich questions	«How would you like to structure the information that you have gathered here now? » (2–2)
14	Varying length of wait time after a student asks a question	The facilitator is asked a question by a student and waits to answer. This has a range of 0 to 1 s. Most of the time the facilitator responds immediately. (3–2).
15	Negotiating whether answers are right or incorrect and why	“I was just wondering one thing. [Name], would you have given this patient oxygen, or would you have waited a little with it, considering that they are a bit over the normal level.” (2–3)
8	Using incorrect or partially correct answers to prompt responses	“Since you mentioned this with the blood becoming acidic, do you think there is a connection with the lactate? » (2–2)
6	Pausing to scan or survey	The facilitator waits and looks at the screen. Afterwards the facilitator says: “So now everyone knows what the Glasgow coma scale is.” (2–3)
1	Eavesdropping on group dialogue	While students are talking, the facilitator shows that she is listening. She responds to it later (2–4)
5	Acknowledge when students demonstrate dialogue	[Name] you want to give oxygen… (1–2)
7	Wait time after a student's response	The facilitator is quiet after a student has given a response. This varies from 0 to 3 s. (2–4)
12	Peer discussion	“Now I'm looking at you a bit [name], but it's clearly open for others to come up with input here. We also think out loud together here..» (4–1)
3	Wait time after a facilitator question	The facilitator asks a question and waits until a student responds. This has a range of 0 to 34 s. An example of 10 s is shown in video (4–1)

Table 5 illustrates the different approaches used. The approaches the facilitator used most are illustrated with two quotes per approach, and the less often used approaches are illustrated with one quote per approach. The adapted coding scheme is presented in Table 4.

### 3.1 Patterns of the most used combination of approaches

We identified patterns in the dialogic teaching by examining the approaches that were used in proximity to each other (Table 6). As Table 6 displays, the most common pattern was approach 10 (“Modelling prompts and body language to encourage continuation”) in combination with approach 10 (“Modelling prompts and body language to encourage continuation”), meaning that the facilitator often used this strategy several times in proximity (440 times, see Table 6). Further, we observed that approach 2 (“Questions linked to resources or tasks”) was used in proximity to approach 16 (“Class management”) 344 times.

**Table 6:** Summary of approaches used in proximity.

Dialogic approaches	1	2	3	4	5	6	7	8	9	10	11	12	13	14	15	16
1	3	1	0	3	0	0	2	1	0	4	2	0	0	1	0	4
2	1	144	50	115	0	4	2	6	21	97	41	0	51	8	10	119
3	0	40	10	65	0	3	0	8	8	34	31	0	24	8	4	55
4	3	219	139	100	0	5	1	5	12	246	28	0	106	20	8	88
5	0	1	0	2	0	0	0	0	0	1	0	0	2	0	0	3
6	1	6	2	9	2	2	0	0	1	10	2	0	5	0	1	5
7	7	1	0	2	0	0	2	0	0	2	0	0	0	0	0	1
8	1	9	8	9	2	3	0	2	1	10	4	0	3	3	3	13
9	1	42	18	43	1	1	0	2	2	28	8	0	15	3	4	16
10	7	96	31	187	2	13	3	8	33	440	58	1	125	20	16	173
11	2	49	48	120	0	3	0	8	7	95	13	0	31	10	5	34
12	0	0	1	0	0	0	0	0	0	0	0	0	0	0	0	1
13	3	90	19	139	3	7	0	11	28	134	34	0	68	6	9	108
14	2	17	1	31	0	1	0	7	5	23	10	0	18	3	3	24
15	1	18	8	13	0	0	0	1	2	20	15	0	8	0	6	10
16	2	344	53	238	1	11	1	20	53	159	81	0	127	42	14	295

## 4 Discussion

This study aimed to adapt an analytic tool to a virtual context, and to explore facilitators' approaches in a virtual simulation setting with virtual patients to conduct reflective dialogue with nursing students. Our findings provide insights into the dialogic approaches and the patterns of approaches facilitators use in dialogic teaching in virtual simulation.

### 4.1 Adaptation of the analytical tool

Our first aim was to adapt the dialogic approaches coding system [22] developed for a traditional classroom setting in primary school to a virtual setting in higher education. The coding system “educational approaches to dialogue” was originally developed for primary education with the aim of making it possible to observe a physical simulation of the dialogical approaches used. The sub-categories used in this study were developed in a previous study by van der Vloed [23]. When using a coding system, it is important to make sure that it is suitable for the specific setting being investigated. We observed that the facilitator approached the simulation session in a way that was not covered in the existing analysis instrument. This resulted in one added sub-code (item 2.4, questions related to the Body Interact program) and one added main code (item 16, Class management) with four sub-codes (16.1 Translation from English to Norwegian, 16.2 Correcting students, 16.3 Class management, and 16.4 Teaching). With these adaptations the coding system was considered suitable for a virtual setting in higher education. According to Alexander [12] all approaches that come under the categories asking, listening, and waiting contribute to promote dialogue regardless of the context. This validates the use of this analysis instrument. The new code 16 that has been developed was mostly observed at the beginning and at the end of the virtual simulations. At these times, an explanation was given about what would be done and the students were asked for approval. The facilitator provided instructions prior to the simulation or concluded the simulation by looking ahead to the next time and instructed students on how they could practice with the simulation program. The approach was also observed throughout the simulations. The facilitator then asked whether the students agreed with her plan and whether the next step in the virtual simulation programme had been taken. A good start through classroom management and a strong ending with a clear goal encourage student engagement [24] and may promote dialogue in the simulation. Class management can also give students a sense of belonging [25], which is a condition for dialogue and learning and was of particular importance for these students, who were not allowed to meet each other in person due to COVID-19 restrictions.

### 4.2 Dialogic teaching approaches in virtual simulation

Our second aim was to explore the approaches used by facilitators in this novel way of conducting simulation sessions. The facilitators in this study most frequently used the approaches categorised under *asking* and *listening*. The most frequent pattern seen in the use of dialogic approaches falls under the category *listening*. By entering a dialogue, facilitators and students can inspire by each other [26,27]. The facilitator is not the centre of the dialogue, but rather searches for subjects of conversation and reflection together with the students. Students can learn from their peers and the facilitator [28,29]. In a virtual simulation with virtual patients, there is also the possibility to learn from the virtual patient or from the software providing feedback, for example through warnings prompts [30], a scoring system [21,31], or direct feedback [32].

Category 10 was most frequently used (“Modelling prompts and body language to encourage continuation”). Typical for this category is that the facilitator encourages students to develop their answers, either by nodding (non-verbal) or by using encouraging phrases, such as “go on…” (oral prompts). The facilitator can also ask another student to elaborate on the answer of a fellow student. By making these prompts explicit the intention is that students will adopt similar strategies in their group dialogue. This is in line with the International Nursing Association of Clinical Simulation and Learning (INACSL) standards for best practice, which state that the role of the facilitator is to assist students' knowledge development by exploring their thought process in critical thinking regardless of the scenario [33]. The standards acknowledge that the facilitator's role differs in in-person and online simulation settings. However, there is no clear description of how virtual facilitation should be executed differently and more research is needed to establish this. The results from this study might contribute to a more precise description of facilitation in a virtual simulation setting.

There were few strategies used in this study that fall under the category *waiting*. This could be because the simulation appointed three students to be active participants while the rest could participate using the chat function, which is less frightening. The fact that the simulations were virtual created a different context for the facilitators. In a physical simulation, the facilitator can use their whole body, as well as the physical space, to encourage students and observe whether a student might want to say something. This is obviously different in virtual simulation. Effective dialogue requires equality [11,12,34], which can be difficult to achieve in a physical room because the facilitator is often situated front and centre. The virtual simulations ensured that everyone was equally visible because all participants were displayed in the same size on the screen. When the facilitator is not the central figure, as in a physical simulation, it might create a different dynamic. The facilitator might also experience less pressure to speak whenever there is silence in the virtual simulation than in a physical simulation, which could explain why we observed a long wait time in some segments after the facilitator asked students. It was observed thirteen times that the facilitator waited longer than 10 s. This occurred mainly after the facilitator asked a student a question. When a student asked the facilitator a question, in almost all cases the facilitator responded immediately with an answer. Nurses are expected to be analytical as well as empathetic. Listening and asking skills are important for this [35,36]. The facilitator was a registered nurse (RN), and it might be considered a strength to have worked in the field and have many real-life examples [37]. It was more difficult for the facilitator to not talk all the time and fill in moments of silence as they would at work. Facilitators often feel the obligation to provide information, and this does not automatically result in more knowledge among the students. Facilitators are responsible for developing space for dialogue [26,38]. The key to success is that the facilitator engages in the dialogue and, like the students, has a voice [5].

Testing the credibility of the data interpretations makes it possible to determine whether the reconstruction of reality, as envisioned by the researchers, is recognizable to the people being observed [39]. In this study, the effect of the dialogical approaches in the virtual simulation was not examined. Reflection in a dialogue is not about getting the right answer but about bringing together the ideas, knowledge, and experience of everyone in the conversation [40]. To gain an in-depth understanding of the simulations and to be able to give meaning to what occurred in the virtual simulations, an extensive conversation with the students could have provided a better picture of the dialogue. Students might have been able to experience dialogue. Examining students' experience and its significance could have strengthened the results.

In the case of this study, it was virtually impossible to view the facilitator's body language and make observations based on this, which may have contributed to the perception that body language indicated whether the facilitator was using dialogical approaches. Not seeing the whole body may affect students' learning [41]. To observe the facilitator, it is necessary to have a good view of the face. On Zoom, the facilitator's face was the size of a passport photograph, and all non-verbal responses might not have been visible. In a few cases, the image disappeared, and the facilitator was not visible. This did not affect the observation of the dialogical approaches used because the facilitator then made herself visible again.

### 4.3 Innovation

This study is innovative in two ways. First, we adjusted a coding system from one setting to apply it to a different setting. Adapting a coding system between settings represents an innovative strategy that enhances the versatility and applicability of research methodologies. Coding systems serve as a framework for organizing and categorizing qualitative data, allowing researchers to derive meaningful patterns and themes. When a coding system is transferred from one setting to another, it encourages collaboration between fields, encouraging the synthesis of knowledge and the development of comprehensive insights that transcend disciplinary boundaries.

Second, we studied a novel simulation setting that not only included virtual patients but was also conducted using a video conferencing system. As the pandemic has forced universities all over the world to provide more virtual learning options, we can study the new practices applied and implement those that seemed to work well. Getting feedback from students is an efficient way for facilitators to learn and change their way of teaching [42]. Students might restrict themselves from giving critical feedback as their facilitator also might be evaluating them in the course. For this reason, objective feedback from research is necessary. The exploration of feedback within virtual simulations is particularly innovative as it allows for analysis on a micro level. This can foster a deeper understanding of the interaction and connection between humans and technologies and contribute to enhancing the effectiveness of training programs. The insights gained from studying feedback in virtual simulations can be applied to education, as well as professional training of healthcare personnel, paving the way for transformative advancements in how we acquire and refine essential skills in an increasingly digital world.

## 5 Conclusion

This research has focused on the use of dialogic approaches in dialogic learning in a virtual educational practice. An important goal of dialogue in education is to stimulate students' learning. We found that the facilitator promoted dialogue by asking and listening. As a next step in applying dialogical approaches, follow-up research can focus on how facilitators can strategically deploy dialogic approaches in other types of simulations with students. It remains to be seen whether students also learn more when facilitators make the dialogue their own. The recommendations from this research can contribute to dialogic teaching in higher education.

## CRediT authorship contribution statement

**Maarten van der Vloed:** Conceptualization, Data curation, Formal analysis, Methodology, Software, Visualization, Writing – original draft, Writing – review & editing. **Hilde Eide:** Conceptualization, Data curation, Funding acquisition, Methodology, Supervision, Writing – review & editing. **Lise Gladhus:** Conceptualization, Data curation, Methodology, Writing – review & editing. **Kirsten Røland Byermoen:** Conceptualization, Validation, Writing – review & editing. **Hugrun Ösp Egilsdottir:** Conceptualization, Validation, Writing – review & editing. **Lena Günterberg Heyn:** Conceptualization, Data curation, Formal analysis, Methodology, Project administration, Supervision, Writing – original draft, Writing – review & editing.

## Declaration of competing interest

The study was partly funded by the Norwegian Directorate of Higher Education.

None of the authors have any conflict of interest to declare.
